# Co-stimulation with equibiaxial strain and pre-osteoblast co-culture differentiates monocytes in a bone model

**DOI:** 10.1557/s43579-025-00711-2

**Published:** 2025-04-17

**Authors:** Maria R. Ward Rashidi, Catherine S. Snyder, Kathleen M. Burkhard, Raneem Ahmad, Isha Bhorkar, Geeta Mehta

**Affiliations:** 1 Department of Materials Science and Engineering, University of Michigan, Ann Arbor, MI 48109 USA; 2 Department of Biomedical Engineering, University of Michigan, Ann Arbor, MI 48109 USA; 3 Department of Chemical Engineering, University of Michigan, Ann Arbor, MI 48109 USA; 4 Macromolecular Science and Engineering, University of Michigan, Ann Arbor, MI 48109 USA; 5 Rogel Cancer Center, University of Michigan, Ann Arbor, MI 48109 USA; 6 AI & Digital Health Innovation, University of Michigan, Ann Arbor, MI 48109 USA

**Keywords:** Biomaterial, Biomimetic, Bone, Functional, Modeling, Morphology, Tissue, Tunable

## Abstract

Bone remodeling and immune function are dynamically regulated through cell–cell and cell–matrix interactions by stem and mature cell populations. We investigated the hypothesis that monocytes and pre-osteoblasts respond to cyclic tensile stress and paracrine interactions by differentiating into macrophage-like and osteoblast-like cells. 20% cyclic equibiaxial strain was applied to monocytic U937 and pre-osteoblastic ST2 cells for 72 h. Increased levels of CD11B, CD14, IL-6, and IL-8 in U937 indicated monocytic differentiation. Increased ALP expression and calcium deposition in ST2 indicated differentiation towards osteoblastic lineage. Overall, application of cyclic strain and pre-osteoblastic co-culture induced differentiation in this cyclically strained bone model.

**Graphical abstract:**

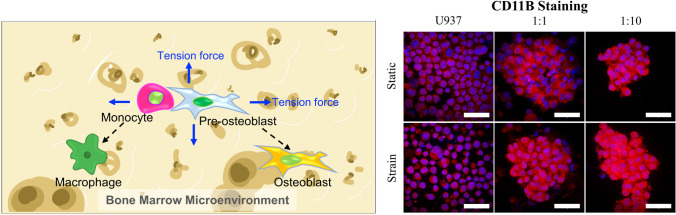

## Introduction

The unique microenvironment of the bone supports the self-renewal and differentiation of several mature and progenitor cell populations. Cell–cell interactions of innate immune cells, such as monocytes and macrophages, with other cell types in the bone regulate homeostasis, differentiation, and remodeling.^[1]^ Adult monocytes, originating from hematopoietic stem cells in the bone marrow, are either released into circulation or retained in the bone marrow to replenish mature monocytes.^[2]^ Bone macrophages also participate in tissue homeostasis, injury repair, immunomodulation, and inflammatory response through multiple distinct resident macrophage populations.^[3]^

Bone tissue is continuously turned over to repair micro-damages. Osteoclasts erode old bone, and osteoblasts produce bone matrix proteins to create new bone in its place. Osteoblasts are derived from multipotent mesenchymal stem cells, which differentiate into pre-osteoblasts and finally osteoblasts in response to various signaling molecules such as bone morphogenic proteins, growth factors, angiogenic factors, and hormones.^[4]^ Additionally, the interaction between monocytes and pre-osteoblasts can promote the formation of osteoclasts, contributing to osteolytic lesions.^[4]^ The spatial heterogeneity of the bone microenvironment determines the biochemical and mechanical cues involved in tissue regulation.

To investigate the impact of paracrine interactions and mechanical stimuli on cellular differentiation in the bone microenvironment, we created a mechanically dynamic *in vitro* model that mimics the crosstalk between pre-osteoblasts (ST2) and monocytes (U937). Bone mass is maintained by continuous input from mechanical stimuli, primarily from muscle contractions occurring throughout daily life.^[5]^ These muscle contractions cause tensile strain and compression in the bone, which also result in fluid shear stress from interstitial fluid exiting the lacuno-canalicular network.^[5]^ Therefore, in this study, we used tension as one of the mechanical stimuli present in the bone to investigate its impact on the interactions between pre-osteoblasts and monocytes in the bone microenvironment.^[4,6]^ We utilized a commercially available tension-stimulating system to apply 20% cyclic equibiaxial strain to the pre-osteoblasts and monocytes, simulating one of the mechanical cues of the bone microenvironment. The magnitude of the equibiaxial tensile strain was chosen based on previous studies on mechanical stimulation of monocytes and osteoblasts.^[5,7]^ Given that the relative number of monocytes and pre-osteoblasts in the human bone tissue are not readily available, we chose two ratios (1:1 and 1:10, U937:ST2) to facilitate co-culture initiated paracrine interactions.^[8,9]^ Moreover, in order to clearly delineate the impact of strain and paracrine interactions in the bone microenvironment, no other chemical cues for differentiation were introduced in the study, such as phorbol 12-myristate 13-acetate (PMA) or lipopolysaccharide (LPS) for monocytes,^[10]^ or osteoblastic differentiation medium for pre-osteoblasts.^[11]^ We reasoned that in the absence of these biochemical cues, we would be able to clearly identify the roles of cyclic strain and paracrine interactions in the bone. Further, we hypothesized that cyclic strain induces differentiation of pre-osteoblasts and monocytes in the bone, priming it towards an inflamed state. This work demonstrates that our mechanically dynamic *in vitro* model can provide an improved understanding of the cellular interactions between osteoblasts and monocytes within the context of the bone microenvironment.

## Materials and methods

### Materials and cells

Reagents used for cell culture were purchased from Thermo Fisher Scientific Inc., unless otherwise mentioned. Cells were cultured in RPMI 1640 cell medium with 10% fetal bovine serum and 1% Antibiotics/Antimycotics and maintained in a 5% CO_2_ humidified incubator at 37℃. The mouse bone-marrow derived pre-osteoblastic stromal ST2 cells and the human myeloid leukemia U937 cells were obtained from ATCC (Manassas, VA). All cells used were below a passage number of 10 for all experiments. BioFlex® 6-well plates with flexible collagen type-1 coated bottom were purchased from Flexcell® International Corporation (Burlington, NC).

### Cyclic tensile strain and co-culture conditions

ST2 and U937 cells were cultured to 70% confluency, trypsinized, counted using a hemocytometer, and appropriately diluted in 3 mL medium to achieve the desired numbers for sub-passaging. Co-culture ratios of 1:1 and 1:10 (U937:ST2), and controls of U937 and ST2 cells alone were plated at a combined total of 150,000 cells per well in a BioFlex® 6-well plate supplied from Flexcell®. ST2 cells were plated on Day 0 to allow for attachment to the BioFlex® well bottom before adding the non-adherent U937 cells on Day 1. Equibiaxial tensile strain of 20% was applied on Day 1 using a FX-4000™ Tension System from Flexcell® International Corporation (Burlington, NC). The strain was cycled at 1 Hertz for 72 h at 20% elongation with static control plates placed within the same incubator but received no strain over the 72 h. Cell medium levels were monitored each day during the 72-h experiment, and any cell medium lost through evaporation was replaced to maintain 3 mL volume. After 72 h, U937 cells and cell medium were collected and centrifuged for 5 min at 300 RCF. Supernatant cell medium was collected, filtered through 20 μm filters, and stored frozen at − 80℃ until used for protein analysis. The U937 cell pellet was resuspended in 1 mL fresh cell medium, and 100 μL of the resuspended U937 cell mixture was cyto-spun onto a glass slide with filter cards (Hettich North America, Beverly, MA), using a cyto-rotor insert in a centrifuge. ST2 cells were left adherent to the BioFlex® well bottoms, and the flexible well bottoms were cut from the plate using a scalpel blade. Both the U937 on glass slides and the ST2 cells on the flexible well bottoms were fixed with 10% neutral buffered formalin and stored in fresh PBS at 4℃ until analysis.

### Immunostaining

U937 cells were stained with CD11B conjugated to Texas Red (Sigma-Aldrich, St. Louis, MO) or FITC-conjugated CD14 (Miltenyi Biotec, Bergisch Gladbach, Germany), with DAPI staining of the nuclei. ST2 cells were stained with either alkaline phosphatase (Vector Laboratories, Burlingame, CA) with DAPI staining of the nuclei, or with the colorimetric Von Kossa stain (Abcam, Cambridge, MA). All immunostaining and mineral staining were done according to the manufacturers’ included protocols.

### Enzyme-linked immunosorbent assay (ELISA)

The ELISA was performed by the University of Michigan Rogel Cancer Center Immunology Core on the saved conditioned medium to quantify interleukin-8 (IL-8) and interleukin-6 (IL-6) levels.

### Imaging

CD11B, CD14, and alkaline phosphatase (ALP) fluorescent images were obtained using an inverted confocal microscope (Olympus IX81, Japan equipped with a Yokogawa CSU-X1 confocal scanning laser, Andor iXon × 3 CCD camera and MetaMorph 7.8 software). Von Kossa images were collected using an EVOS XL Core Microscope (Thermo Fisher Scientific Inc).

### Quantification and statistical analysis

All immunofluorescent and other images were quantified using Image J (National Institutes of Health). Fluorescence intensities of the CD11B, CD14, and alkaline phosphatase images were normalized to the corresponding intensity of DAPI. Von Kossa images were analyzed for calcium coloration and normalized to the number of cells in each image. All quantifications had an *N* of 7 or more, and data analyses were performed and are reported as the mean with error bars representing the standard error of the mean (SEM). Significance between groups was assessed using a two-tailed student’s *t*-test and considered significant if p < 0.05. Significant differences are indicated with the following symbols: “*” indicates significance between the static and strained conditions of the same co-culture, “‡” indicates significance between the value and the corresponding ST2 co-culture of the same mechanical condition, “#” indicates significance between the denoted value and the 1:1 (U937:ST2) co-culture with the same mechanical condition, “†” indicates significance between the denoted value and the 1:10 (U937:ST2) co-culture with the same mechanical condition and “§” indicates significance between the denoted value and all other co-cultures of the same mechanical condition.

## Results and discussion

### Co-culture and cyclic strain activate monocyte to macrophage differentiation in U937 cells

**Figure 1:**
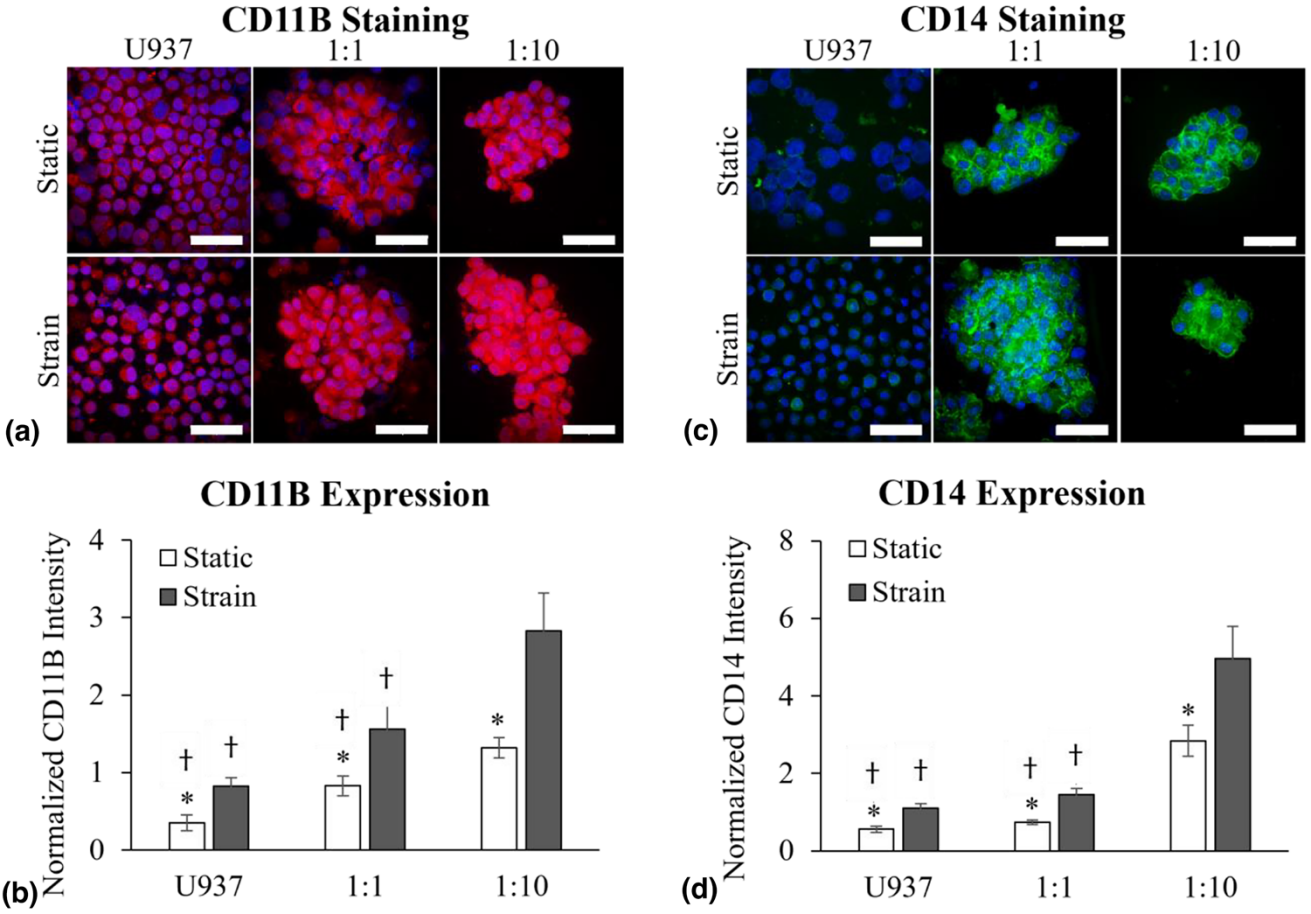
Monocytic activation and differentiation under cyclic strain and co-culture with pre-osteoblasts were quantified by changes in CD11B and CD14 levels in U937 cells after 72 h of co-culture with ST2 and/or 20% cyclic strain exposure. Fluorescent confocal images show U937 cells stained with (a) CD11B (red), (c) CD14 (green) and DAPI-nuclei (blue). The scale bar is 50 µm. Quantification of (b) CD11B and (d) CD14 expression was determined from fluorescent confocal images after normalization to DAPI intensity. Significance was determined with paired *t*-tests where p < 0.05. “*” indicates significance between the static and corresponding strain condition of the same experimental condition and “†” indicates significance between the denoted value and the 1:10 (U937:ST2) co-culture of the same mechanical stimulation condition.

We investigated monocytic differentiation in the bone microenvironment by quantifying two common biomarkers of monocyte to macrophage differentiation, CD11B and CD14, in U937 cells using quantitative immunofluorescence (IF). Both CD11B^[12]^ and CD14^[13]^ have been previously established as markers of U937 macrophage differentiation using chemical stimulants such as vitamin D3 or phorbol-12-myristate-13-acetate (PMA). Figure 1(a) depicts U937 cells with stained nuclei (blue) and CD11B protein (red) across all experimental conditions in both static control and 20% cyclic strain environments. A noticeable increase in the amount of CD11B, a surface adhesion molecule upregulated during monocytic differentiation, was observed in the 1:1 (U937:ST2) and 1:10 (U937:ST2) co-cultures, as compared to the U937 only controls. Additionally, increased clustering of U937 cells was observed with co-culturing, where the 1:1 (U937:ST2) displayed loose clusters, and the 1:10 (U937:ST2) displayed very tight cell clusters. Figure 1(b) shows the quantification of the fluorescent confocal microscopy images where the amount of CD11B (red intensity) is normalized to the number of cells (DAPI intensity). Significant increases in CD11B levels were observed in the cyclic strain conditions compared to static, regardless of the presence of ST2 cells. U937 alone exhibited a 2.35-fold increase in CD11B under cyclic strain. When co-cultured with pre-osteoblastic ST2 cells, U937 cells demonstrated a 1.88-fold and a 2.14-fold (1:1 and 1:10 (U937:ST2)) increase in CD11B levels with the application of cyclic strain, respectively. In the absence of cyclic strain, CD11B levels increased, though not significantly, in the 1:1 (U937:ST2) compared to the U937 monoculture with a fold change of 2.37. However, CD11B was significantly higher in the 1:10 (U937:ST2) compared to both the U937 monoculture (3.77-fold increase) and the 1:1 (1.59-fold increase), respectively. Under cyclic strain conditions, the same trend was observed as in the static conditions, with no significant difference between the U937 monoculture and the 1:1 (U937:ST2) co-culture, showing a fold change of 1.90. However, in the 1:10 and the 1:1 (U937:ST2) conditions, there was a significant increase in CD11B, with fold changes of 3.44 and 1.81, respectively, compared to U937 monoculture. Therefore, the cell–cell interactions between ST2 and U937 under cyclic equibiaxial strain produced significantly enhanced differentiation of monocytes. Although an increase in CD11B is generally associated with increased cell adhesion,^[14,15]^ we did not see a difference in cell–cell adhesion in our confocal microscopy images between the static and strained conditions of the monoculture [Fig 1(a)].

Similarly, CD14 (green), a cell surface marker for bacterial lipopolysaccharide expressed by macrophages, was quantified under cyclic strain and co-culture paracrine interaction conditions, as shown in Fig 1(c). Increased clustering of the U937 cells and elevated levels of CD14 were observed when U937 cells were co-cultured with ST2 cells. The quantification of CD14 expression (green fluorescence integrated intensity) normalized to the number of cells (DAPI intensity) is shown in Fig 1(d). There was a significant increase in CD14 levels with applied cyclic strain compared to static in all culture conditions. With the application of cyclic strain, the U937 monoculture displayed a 1.96-fold increase in CD14 level, the 1:1 (U937:ST2) co-culture displayed a 1.95-fold increase, and the 1:10 (U937:ST2) displayed a 1.74-fold increase. Co-culturing U937 cells with ST2 cells under static conditions resulted in significant changes in CD14 levels, with the 1:10 (U937:ST2) co-culture showing a 5.08-fold increase compared to the U937 monoculture and a 3.83-fold increase compared to the 1:1 (U937:ST2) co-culture. When cyclic strain was applied, a similar trend was observed, with the 1:10 (U937:ST2) co-culture expressing 4.53-fold more CD14 than the U937 monoculture and 3.42-fold more than the 1:1 (U937:ST2) co-culture. No significant change in CD14 level was observed for either static or cyclic strain conditions when comparing the U937 monoculture to the 1:1 (U937:ST2) co-culture, with fold changes of 1.33 and 1.32 for static and cyclic strain conditions, respectively. These data collectively indicate that U937 monocytes initiate a differentiation cascade towards macrophages, with increased CD14 and CD11B levels, under the influence of mechanically active co-cultures with pre-osteoblasts.

### Secretion of macrophage associated inflammatory cytokines IL-6 and IL-8 in U937 cells increases with the application of cyclic strain and co-culturing with ST2 cells

**Figure 2:**
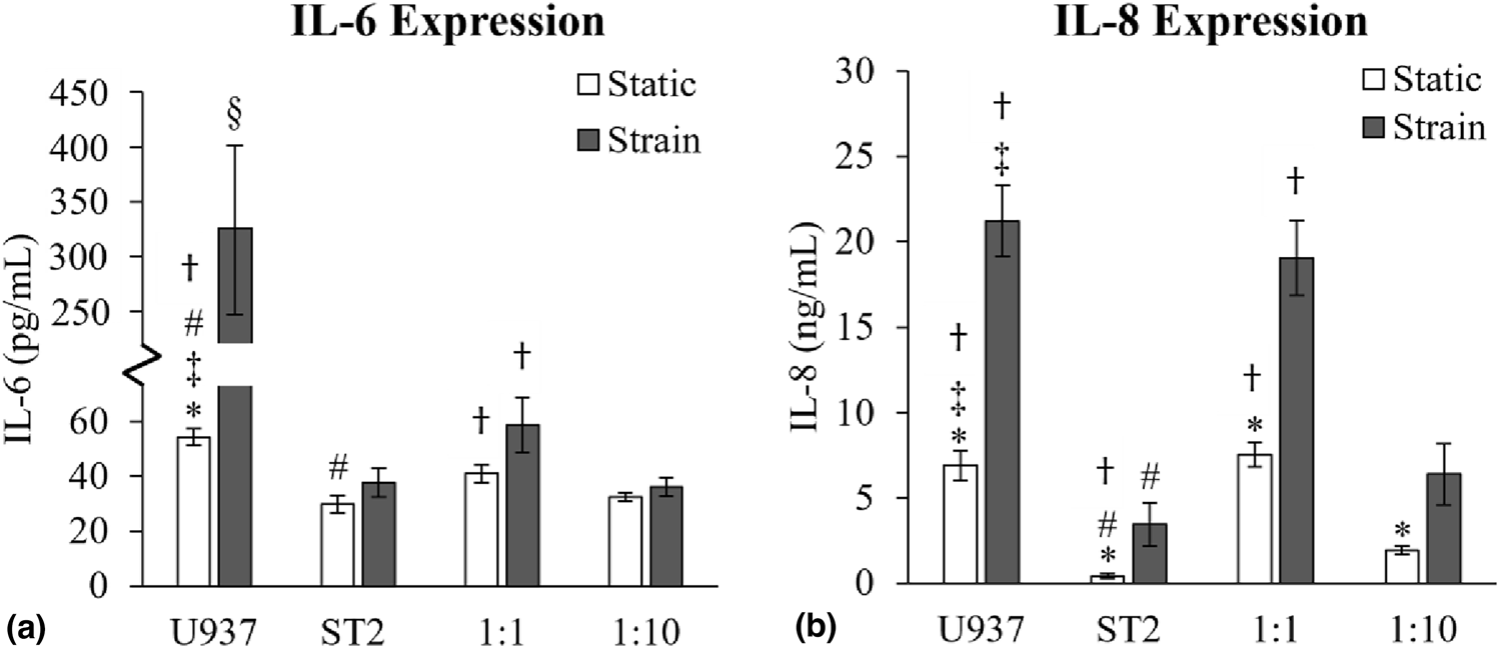
Macrophage-associated inflammatory cytokine protein expression changes with the application of 20% cyclic strain and/or pre-osteoblast co-culture. (a) IL-6 and (b) IL-8 expression levels in the cell medium, quantified with an ELISA. N = 8. (b) Significance was determined with a *t*-test where p < 0.05. “*” indicates significance between the static and corresponding strain condition of the same culture condition, “‡” indicates significance between the value and the corresponding ST2 co-culture of the same mechanical condition, “#” indicates significance between the denoted value and the 1:1 (U937:ST2) co-culture of the same mechanical stimulation condition, “†” indicates significance between the denoted value and the 1:10 (U937:ST2) co-culture of the same mechanical stimulation condition, and “§” indicates significance between the denoted value and all other co-cultures of the same mechanical condition.

IL-6 and IL-8 secretion are hallmarks of monocyte differentiation that increase with the addition of vitamin D3 or PMA treatment *in vitro*.^[10,16]^ IL-6 and IL-8 signaling is also involved in osteoclastogenesis.^[17]^ Since differentiated macrophages produce these inflammatory cytokines, we quantified levels of IL-6 and IL-8 in the cell culture medium using an enzyme-linked immunosorbent assay (ELISA) (Fig 2). Monocytic U937 monocultures produced significantly more IL-6 under cyclic strain (328 ± 81 pg/mL) compared to static conditions (54 ± 3 pg/mL) [Fig 2(a)]. In contrast, since pre-osteoblasts do not typically secrete proinflammatory cytokines associated with osteolysis, IL-6 and IL-8,^[18,19]^ no significant difference in IL-6 production was observed in ST2 monoculture between static (30 ± 3 pg/mL) and cyclic strain (38 ± 5 pg/mL) conditions. As expected, IL-6 levels in the U937 culture were significantly higher than that in ST2 culture under both the static and cyclic strain conditions. IL-6 expression in the static (41 ± 3 pg/mL) and cyclic strained (59 ± 10 pg/mL) 1:1 (U937:ST2) co-culture was significantly lower than in the corresponding U937 monoculture conditions. There was no significant difference in IL-6 levels between the static and cyclic strain conditions in the 1:1 or the 1:10 (U937:ST2) co-cultures. The static 1:10 (U937:ST2) co-culture had an IL-6 level (32 ± 2 pg/mL) significantly lower than the static U937 monoculture and the static 1:1 (U937:ST2) co-culture. Similarly, with the application of cyclic strain, the 1:10 (U937:ST2) co-culture IL-6 level (36 ± 3 pg/mL) was significantly lower than both the U937 monoculture and 1:1 (U937:ST2) co-culture under cyclic strain.

Expression of IL-8, quantified by ELISA, is shown in Fig 2(b). The U937 monoculture significantly increased IL-8 production under cyclic strain (21.2 ± 2.1 ng/mL) compared to the static control (6.9 ± 0.9 ng/mL). Since ST2 do not secrete IL-8,^[18,19]^ their monoculture produced only 0.4 ± 0.2 ng/mL of IL-8 under static conditions, increasing to 3.4 ± 1.3 ng/mL under cyclic strain. In both static and cyclic strain conditions, U937 cells produced significantly more IL-8 than ST2 cells. The 1:1 (U937:ST2) and 1:10 (U937:ST2) co-cultures also both produced significantly more IL-8 when cyclic strain was applied (19.0 ± 2.2 ng/mL and 6.4 ± 1.8 ng/mL, respectively) compared to their static controls (7.5 ± 0.7 ng/mL and 1.9 ± 0.3 ng/mL, respectively). In the static 1:1 (U937:ST2) co-culture, IL-8 levels were not significantly different from the static U937 monoculture but were significantly higher than both the static ST2 culture and the static 1:10 (U937:ST2) co-culture. This trend was also observed under cyclic strain conditions. Similarly, in the static 1:10 (U937:ST2) co-culture, IL-8 levels were significantly lower than those in the static U937 monoculture and the static 1:1 (U937:ST2) co-culture, yet significantly higher than in the static ST2 culture. This same pattern was seen under cyclic strain conditions.

While the strained U937 cells exhibit an increased macrophage-like phenotype, previous research has shown that U937 cells treated with PMA become adherent and increase IL-6 expression when subjected to 10% biaxial strain.^[20]^ Additionally, fluid flow currents generated within the *in vitro* dynamic microenvironment model also exposed the U937 cells to shear stress. This fluid shear stress is physiologically relevant, as loading and unloading of the bone causes interstitial fluid to flow through the pericellular matrix, creating shear forces. Immune cells, such as monocytes and macrophages respond to mechanical stimuli like tension, compression, and shear forces *via* ion channels and other membrane sensors.^[21–23]^ Furthermore, the tensile strain explored in previous studies on macrophages is similar to that in our *in vitro* bone microenvironment model.^[21]^ Since both shear and tension are critical for the bone microenvironment, monocytes were activated and differentiated by the tensile and shear stresses, even in the absence of pre-osteoblasts.

### Early- and late-stage markers of osteoblast differentiation, ALP and calcium mineralization, increase with U937 coculture and the application of cyclic strain in ST2 cells

**Figure 3:**
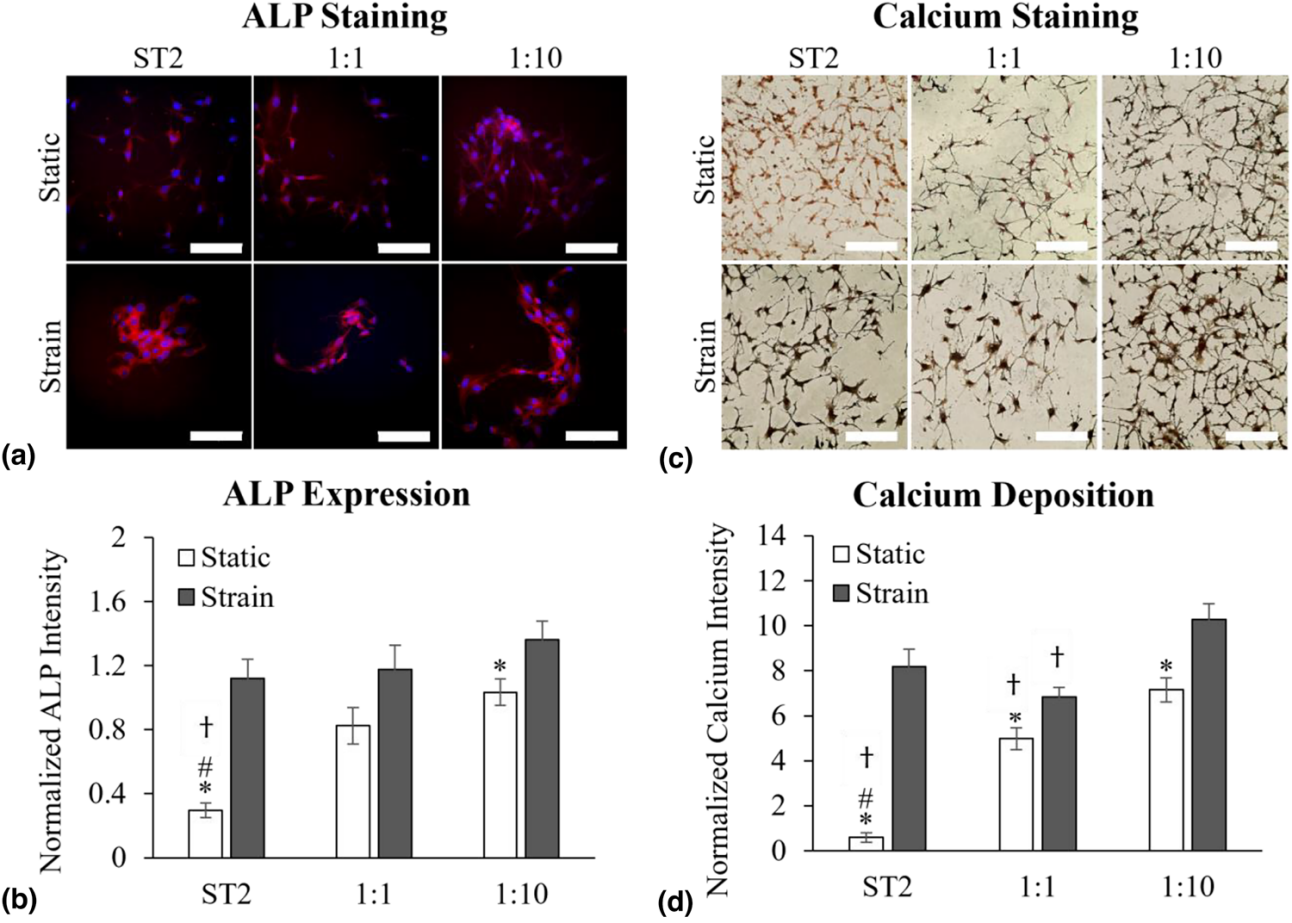
Changes in osteoblast differentiation markers, alkaline phosphatase (ALP) expression and calcium deposition, in ST2 cells following 72 h of co-culture with monocytes and/or the application of 20% cyclic strain. (a) Fluorescent confocal images of ST2 cells stained with ALP (red) and DAPI-nuclei staining (blue). The scale bar is 100 µm. (b) Quantification of ALP expression from confocal images (a), normalized to DAPI intensity. N > 8. (c) Color images of calcium deposition (black) of ST2 cells (red nuclei staining) imaged with an optical microscope. The scale bar is 200 µm. (d) Quantification of calcium deposits using a Von Kossa stain (c), normalized to the number of cells. N > 7. Significance was determined with a t-test where p < 0.05. “*” indicates significance between the static and corresponding strain condition of the same culture condition, “#” indicates significance between the denoted value and the 1:1 (U937:ST2) co-culture of the same mechanical stimulation condition, and “†” indicates significance between the denoted value and the 1:10 (U937:ST2) co-culture of the same mechanical stimulation condition.

Increased alkaline phosphatase (ALP) expression is recognized as an early-stage differentiation marker of osteoblast-like behavior.^[24,25]^ Calcium mineralization is a late-stage differentiation marker of pre-osteoblast to osteoblast differentiation.^[25]^ Therefore, we utilized ALP activity and calcium deposition to assess the extent of differentiation of pre-osteoblastic ST2 into osteoblasts. After 72 h in culture, ALP expression was imaged using confocal microscopy, as shown in Fig 3(a). ALP expression (red fluorescence) increased with cyclic strain, and clustering with limited orientation of ST2 cells (indicated by DAPI nuclei staining) was observed in the U937 co-culture and cyclic strain conditions. Quantification of ALP expression from the confocal images is presented in Fig 3(b), where ALP fluorescence intensity is normalized by the number of DAPI-positive cells. A significant increase in ALP levels was observed with the application of cyclic strain in the ST2 monoculture and the 1:10 (U937:ST2) co-culture, with increases of 3.77-fold and 1.32-fold, respectively. However, cyclic strain in the 1:1 (U937:ST2) co-culture did not result in a significant difference compared to the static control. Under static conditions, ALP levels in ST2 cells significantly increased when co-cultured with U937 cells, showing a 2.78-fold increase in the 1:1 co-culture and a 3.48-fold increase in the 1:10 co-culture. The increase in ALP levels from the 1:1 to the 1:10 co-culture ratio under static conditions was not significant. Although cyclic strain increased ALP levels across all ST2 experimental groups, co-culture with U937 cells did not result in a significant difference in ALP levels under these cyclic strain conditions. Previous studies have demonstrated that applying mechanical strain alongside a chemical additive can increase ALP expression in ST2 cells.^[26]^ However, ST2 cells in our *in vitro* dynamic bone microenvironment model displayed an increase in ALP and calcium mineralization without a chemical differentiation stimulant, suggesting that ST2 cells can initiate differentiation towards osteoblasts solely through the application of mechanical strain.

Calcium mineralization, a late-stage marker of osteoblast differentiation,^[27]^ was quantified using Von Kossa staining after 72 h in culture. Representative optical microscopy images of mineralization in ST2 cells (red nuclei) are presented in Fig 3(c). Increased calcium (black) is seen with the application of cyclic strain. Additionally, co-culturing with U937 cells increased the visible amount of calcium mineralization, especially in the 1:10 (U937:ST2) co-culture. Figure 3(d) presents the quantification of the Von Kossa staining where the amount of calcium (black intensity) is normalized to the number of cells. Calcium production significantly increased in all culture conditions with the application of cyclic strain, showing 13.79-fold, 1.37-fold and 1.44-fold increases for the individual ST2 culture, the 1:1 (U937:ST2) co-culture, and the 1:10 (U937:ST2) co-culture, respectively. Under static conditions, co-culturing ST2 cells with U937 cells led to a significant increase in calcium deposition. There was an 8.41-fold increase between the static ST2 monoculture and the 1:1 (U937:ST2) co-culture, and a 12.08-fold increase between the ST2 monoculture and the 1:10 (U937:ST2) co-culture. The difference between the two static co-culture conditions was also significant. While cyclic strain elevated ST2 calcium deposition, it was significant only when comparing the 1:1 (U937:ST2) to the 1:10 (U937:ST2) co-culture. Under cyclic strain, neither co-culture showed significantly different ALP levels compared to the ST2 monoculture.

Examining the two coculture conditions, significantly higher expression of CD11B and CD14 was observed with a co-culture ratio of 1:10 (U937:ST2), indicating that monocytes in the bone can differentiate into macrophages when exposed to pre-osteoblastic stromal cells. Confocal images of U937 cells stained for CD11B and CD14 also demonstrate increased cell–cell adhesion with co-culture suggesting that the presence of ST2 cells leads to a more adherent phenotype in the U937 cells. Additionally, co-culture of U937 cells and ST2 cells resulted in a significant increase in IL-6 production, particularly in the 1:10 (U937:ST2) co-culture condition, with no difference between static and strained conditions. This increase in IL-6 expression is attributed to the presence of ST2 cells based on previous observations of bone marrow stromal cells (BMSCs) co-cultured with bone marrow derived macrophages initiating a positive autocrine feedback loop of IL-6 production by the macrophages.^[14]^ The greatest increase in ALP expression was observed in the 1:10 (U937:ST2) co-culture condition, however this increase was not significant compared to the 1:1 (U937:ST2) co-culture condition. Co-culturing ST2 cells with U937 cells is thought to induce ST2 differentiation through the secretion of TGF-β by the U937 cells. This co-culture significantly increased calcium deposition in the 1:10 (U937:ST2) condition resulting in higher calcium accumulation than in the 1:1 (U937:ST2) co-culture. When cyclic strain was combined with co-culture conditions, the largest increases in CD11B and CD14 markers in U937 cells were observed in the strained, 1:10 (U937:ST2) co-culture ratio. This suggests that both cyclic strain and co-culture caused activation and differentiation of monocytes towards macrophages. It is currently unknown whether mechanical strain and co-culture induce alternate differentiation pathways or if they act synergistically to produce a larger phenotypic change in the U937 cells. However, we have demonstrated that non-chemical stimuli, similar to those present in the bone environment, can differentiate monocytes to become more macrophage-like. Moreover, the application of strain resulted in the greatest increases in ALP expression and calcium deposition compared to co-culturing with U937 cells, highlighting the importance of mechanical stimulation in studies of the bone niche.

The mechanically dynamic bone microenvironment model presented here could be utilized in investigations of the bone under homeostasis, or pathological conditions such as osteoporosis. Mechanical loading is critical to the regulation of bone tissue, and monocytes secrete cytokines that contribute to bone loss in osteoporosis, though these mechanisms are not fully understood.^[27]^ Since the creation of pre-metastatic niches in the bone and bone marrow includes osteoblasts, osteoclasts, immune cells and other supportive cell populations as key players, this model could potentially be used to study molecular mechanisms involved in cancer metastasis to bone, particularly in metastatic breast cancer.^[28,29]^

## Conclusion

**Figure 4:**
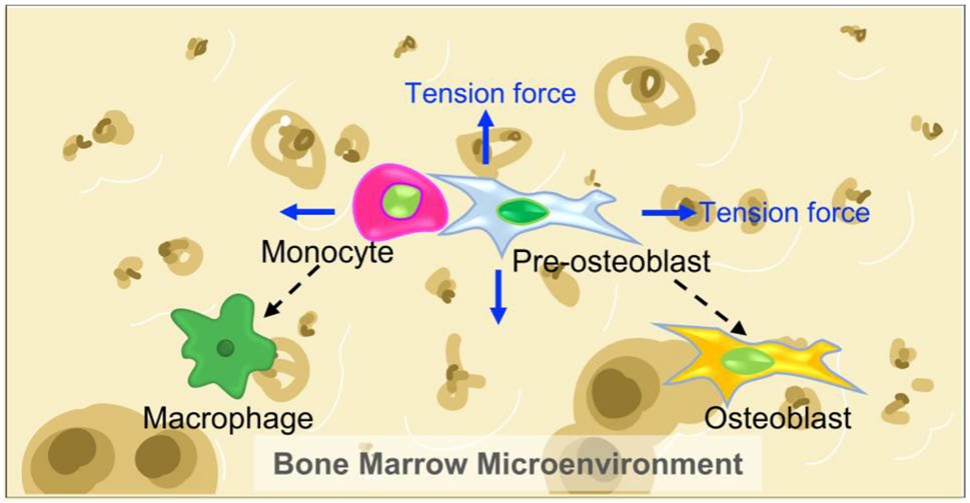
Schematic depicting application of tensile stress to monocytes and pre-osteoblasts in the bone microenvironment. Undifferentiated monocytes and pre-osteoblasts respond to cyclic tensile stress and paracrine interactions by differentiating into macrophage-like and osteoblast-like cells.

In this short report, we developed an *in vitro* mechanically dynamic model representing a simplified microenvironment in the bone, to investigate cell–cell interactions under mechanical strain (Fig 4). An equibiaxial cyclic tensile strain was applied to cell lines representing monocytes and pre-osteoblasts in the bone. Paracrine interactions between the two cell types, U937 monocytes and ST2 pre-osteoblasts, were explored at ratios 1:1 or 1:10 (U937:ST2). Our data demonstrated that cyclic tensile strain, along with the presence of U937 monocytes, without chemical additives, induced an osteoblast-like phenotype in ST2 cells after 72 h, as evidenced by increased ALP expression and calcium mineralization. Additionally, tensile strain directed the activation and differentiation of non-adherent monocytes as confirmed by increased levels of CD11B, CD14, IL-6, and IL-8. Combinations of co-culture and cyclic tensile strain resulted in the highest levels of expression of CD11B and CD14 in U937 monocytes and the highest, albeit not significantly highest, expression levels of ALP and calcium in ST2 cells. These results suggest that the methods employed in this study create a mechanically informed model to study the bone microenvironment *in vitro*. This *in vitro* bone model can be further tuned to investigate mechanical stimulation and cell–cell interactions within the bone microenvironment in both healthy and diseased states.

## Data Availability

The data that support the findings of this study are available from the corresponding author upon reasonable request.

## Abbreviations

ALP: Alkaline Phosphatase
BMSCs: Bone Marrow Stromal Cells
ELISA: Enzyme Linked Immunosorbent Assay
IHC: Immunohistochemistry
IL-6: Interleukin-6
IL-8: Interleukin-8
SEM: Standard Error of the Mean
PMA: Phorbol-12-myristate-13-acetate
